# Provision of domiciliary dental service to residential aged care facilities: A 3‐year descriptive summary

**DOI:** 10.1111/scd.12912

**Published:** 2023-08-07

**Authors:** Kuang‐Yin Chu, Frederick Allan Clive Wright, Vasi Naganathan, Fiona Stanaway, Juliette Tran, Deborah Cockrell

**Affiliations:** ^1^ Centre for Education and Research on Ageing Department of Geriatric Medicine Concord Repatriation General Hospital Sydney Local Health District Concord Australia; ^2^ Concord Clinical School Faculty of Medicine and Health University of Sydney Sydney Australia; ^3^ School of Public Health University of Sydney Sydney Australia

**Keywords:** aged care, domiciliary service, oral health, residential aged care

## Abstract

**Aim:**

This cross‐sectional descriptive study described the oral health status and types of domiciliary dental treatment received by residents living in residential care after an oral health assessment (OHAT).

**Methods:**

Twenty‐one facilities were recruited where consenting participants received OHAT followed by a referral for further domiciliary dental treatments. Data were captured and stored as Reach‐OHT database where 2017–2019 data were analyzed.

**Result:**

Overall, 88% of residents consented. 69.1% were referred for treatment after completion of OHAT. More than half had one or more caries; 40% showed sign of periodontal disease; a higher proportion of dentate participants had an unsatisfactory level of oral cleanliness. Of those received domiciliary dental treatment, diagnostic and preventive service was the combination most frequently provided. These comprised an average of 71.9% of total treatment provided across the 3‐year period.

**Conclusion:**

This study contributes to the understanding and knowledge around the provision of domiciliary dental services in residential care. A large number of older people in residential care can be assessed and treated through a domiciliary service pathway. As the vast majority of services provided were diagnostic, preventive, and restorative care, the feasibility of utilizing the skillset of the entire dental team should be explored.

## 1 INTRODUCTION

Older people in residential aged care facilities have poorer oral health than their community‐dwelling peers.^1^, ^2^, ^3^, ^4^ There are several reasons for this but one of the most important reasons is limitation of access to oral health care. The 2012 New Zealand Older People's Oral Health Survey reported that only one in six older people living in residential care had visited a dental practitioner for a check‐up in the previous 12 months.^5^ In a study by Gerritsen *et al* examining the treatment needs of residents aged over 70 years living in nursing homes, it was reported that the majority (78%) of short‐term residents were ‘in arrears’ with dental treatment. All dentate residents, with the exception of one long‐term resident, needed treatment.^6^ As more natural teeth are retained, expectations for function and aesthetics for older people are increasing. Function, including the ability to eat and taste a wider food selection, and the ability to talk and smile freely, are valued and ranked highly on quality of life scales amongst older people.^7^, ^8^ The unmet demands and the higher prevalence of disorders in the mouths of older people in residential care may indicate that the dental treatments available to residents are insufficient.^9^, ^10^

In Australia there are few fixed dental clinics established in residential aged care facilities. One of the identified strategies to alleviate such inadequacy is to implement a domiciliary service model of care. Domiciliary service is defined as a service put in place to support an individual in their own home, in this instance, residential aged care facilities. This minimizes or removes barriers to those in residential accommodation who are not able to access care in a traditional dental setting.^11^ There are a growing number of public and private operated domiciliary dental services in Australia; however, little is described in the literature on the type of services provided. One of the limitations to providing this model of care is getting adequate resources for dental practitioners to assess and treat patients in this setting. In our local health district, we established a dental domiciliary service program (*Reach‐OHT*) in 2013, to provide dental services to several residential care facilities.^12^ This model of care provided oral health assessments (OHAT) by oral health therapists and, uniquely, dental treatment by private dentists. In Australia, the majority of dentists work as private providers. As part of *Reach‐OHT*’s operation, a database was established to collect data on the oral health status of older people referred for dental treatment and the type and frequency of domiciliary dental services prescribed by dental practitioners.

The aim of this study was to describe the oral health status and types of dental treatment received by residents living in residential aged care facilities who were referred to a dentist after an OHAT by an oral health therapist. This cross‐sectional descriptive study analyzed de‐identified data from the 2017–19 *Reach‐OHT* database.

## 2 METHODOLOGY

*Reach‐OHT*, is a public‐funded dental domiciliary service program which operates from Concord Repatriation General Hospital, Sydney Local Health District (SLHD), Australia. It is governed by a panel of field experts in geriatric medicine and oral health, policy makers, and consumer representatives.^12^ *Reach‐OHT* is coordinated by oral health therapists. Its services include OHATs, education & training of residential care staff by the oral health therapists and dental treatment by dentists. Each participating facility receives a block‐schedule of services annually; additional review can be requested episodically to see residents with more acute problems.^12^

Twenty‐one participating facilities were recruited from quarterly district residential aged care nursing meetings. All residents living in the participating facilities, their families (or legal guardians) were approached and invited to join the program. Informed consent was obtained from individual residents, or their legal guardians. Each consenting participant received an OHAT provided by calibrated oral health therapists. OHAT is a component of the Best Practice Oral Health Model for Australian Residential Care study that provides institutional carers with a simple, eight category screening tool to assess resident's oral health (Appendix 1).^13^ Based on the OHAT outcomes, a referral for further dental treatment was made to participating private dentists. Decision for referral took into consideration the participant's physical and cognitive status; behavioral complications; consent; and the oral health parameters prescribed in OHAT. Participating dentists were recruited following an information session on domiciliary dental services hosted by *SLHD*. Dentists were then assigned to RACFs based on their proximity to the RACFs and their work schedules. To proceed with further dental treatment, a separate consent to the initial assessment consent was required. A range of portable dental equipment was provided by the *Reach‐OHT* program. A list of this equipment is provided in Table 1. Dentists were reimbursed for the services they provided through an established Fee for services scheme through NSW Health. All the services provided by the dentists during the 3‐year period were itemized and categorized using the Australian Government Department of Veterans’ Affairs Schedule of Fee for Dental Services guidelines (Appendix 2).

**TABLE 1:** List of portable equipment provided by Reach‐OHT.

Equipment
1. Mobile dental delivery unit
2. Reclinable wheelchair
3. Handheld x‐ray unit
4. Mobile suction unit
5. Portable light
6. Dentist and assistant stool
7. Digital x‐ray scanner
8. Laptop
9. Electric handpieces
10. Ultrasonic scaler handpieces and tips
11. Instrument cart
12. Diagnostic consumables
13. Preventive consumables
14. Restorative consumables

Data for this cross‐sectional study was from the OHAT and dental treatment database used for the *Reach‐OHT* program operations and was from the period of 2017 to 2019. It is important to note that we are describing the cohort that was seen each year and many participants were seen in more than 1 year. Approval to analyze data from the database was obtained through Sydney Local Health District Human Research Ethics Committee—CRGH (LNR/18/CRGH/166). Protection of study participants’ privacy were fulfilled by meeting the requirements set by The NSW Health Privacy and Confidentiality protocol where all personal information was de‐identified in data analysis and for publication purposes.^14^ The de‐identified database was stored electronically in a secure server within the Department of Geriatric Medicine and Rehabilitation Medicine, Concord Repatriation General Hospital. Access to information was only available to the primary investigator of this study who is also the *Reach‐OHT* program coordinator. Descriptive statistical analysis was completed using IBM SPSS Statistics version 21. Data are presented as means or frequency and percentages for continuous and categorical variables, respectively.

## 3 RESULTS

### 3.1 Participants’ demographics

The number of residents included in this analysis are summarized in Table 2. Overall, 88% of residents approached consented to have an OHAT completed. Data on all of these residents were available in the database. The mean age of participant was 86 years (SD = 8.8) and the largest number of participants were between 85 and 94 years‐old (51.0%). Amongst the total number of participants, 68.0% were female.

**TABLE 2:** Age and gender distribution of the participants in each calendar year.^a^

Year	2017	2018	2019	Total
Residents approached	854	1225	1444	3523
Consent to OHAT				
Consented	752 (88.1)	1090 (89.0)	1259 (87.2)	3101 (88.0)
Declined	102	135	185	422
Mean age (years)	86.15	85.72	86.18	86.01
Age group				
≥64	16 (2.1)	31 (2.8)	35 (2.8)	82 (2.6)
65–69	13 (1.7)	35 (3.2)	32 (2.5)	80 (2.6)
70–74	32 (4.3)	50 (4.6)	56 (4.5)	138 (4.5)
75–79	70 (9.3)	92 (8.4)	108 (8.6)	270 (8.7)
80–84	150 (20.0)	203 (18.6)	208 (16.5)	561 (18.1)
85–89	191 (25.4)	268 (24.6)	312 (24.8)	771 (24.9)
90–94	188 (25.0)	279 (25.6)	342 (27.2)	809 (26.1)
>95	92 (12.2)	132 (12.1)	166 (13.2)	390 (12.6)
Gender				
Male	237 (31.5)	348 (31.9)	407 (32.3)	992 (32.0)
Female	515 (68.5)	742 (68.1)	852 (67.7)	2109 (68.0)

*Note*: Value are numbers (percentages) unless otherwise stated.

^a^ Participants were able to receive services provided by Reach‐OHT each calendar year.

### 3.2 Referrals and completion rate

Of those who completed the OHAT, 2143 (69.1%) were referred to dentists for further treatment. Of those referred, 1398 (65.2%) completed the proposed treatments (Table 3). There were various reasons why participants declined treatments, including behavioral complications, having their own dentist, a preference to receive treatment in a hospital setting, or electing to have palliative care only.

**TABLE 3:** Proportion of participants referred to dentists and proportion who completed the course of care proposed by dentists.

	2017	2018	2019	Total
Assessed by oral health therapists	752	1090	1259	3101
Referred to dentists	477 (63.4)	772 (70.8)	894 (71.0)	2143 (69.1)
Referred and completed the proposed treatment	336 (70.4)	518 (67.1)	544 (60.9)	1398 (65.2)

*Note*: Value are numbers (percentages) unless otherwise stated.

### 3.3 Oral health status of the participants referred to dental practitioners

The oral health status of the participants referred for further dental treatment are described in Table 4. Notably, more than two thirds of those referred did not have sufficient cognitive or physical capacity to practice effective daily oral hygiene and more than three quarters of the participants were physically dependent on others for assistance with daily activities. A higher proportion (48.2%–71.3%) of dentate participants seen each year had an unsatisfactory level of oral cleanliness. More than half had one or more carious lesions present and more than 40% showed signs of periodontal disease. Only a minority reported or showed physical signs of dental‐related pain (6.1%, 7.0%, and 12.4%).

**TABLE 4:** Oral health status based on OHAT of the participants referred to dentists.

Year	2017	2018	2019
Number referred	477	772	894
Ability to practice daily oral hygiene			
Independent	165 (34.6)	221 (28.6)	280 (31.3)
Dependent	312 (65.4)	551 (71.4)	614 (68.7)
Physical condition			
Independent	94 (19.7)	181 (23.4)	181 (20.2)
Dependent	383 (80.3)	591 (76.6)	713 (79.8)
Level of communication			
Able to communicate effectively	308 (64.6)	512 (66.3)	633 (70.8)
Unable to communicate effectively	169 (35.4)	260 (33.7)	261 (29.2)
Behavioral complication			
No behavioral complication	435 (91.2)	692 (89.6)	783 (87.6)
Difficult behavioral complications present	42 (8.8)	80 (10.4)	111 (12.4)
Lips			
Healthy	422 (88.5)	612 (79.3)	776 (86.8)
Changed/Unhealthy	55 (11.5)	160 (20.7)	118 (13.2)
Tongue			
Healthy	366 (76.7)	502 (65.0)	631 (70.6)
Changed/Unhealthy	111 (23.3)	270 (35.0)	263 (29.4)
Gum & soft tissues			
Healthy	282 (59.1)	357 (46.2)	484 (54.1)
Changed/Unhealthy	195 (40.9)	415 (53.8)	410 (45.9)
Saliva			
Healthy	324 (67.9)	427 (55.3)	584 (65.3)
Changed/Unhealthy	153 (32.1)	345 (44.7)	310 (34.7)
Natural teeth			
No decay present	205 (45.5)	295 (41.8)	311 (38.1)
Decay(s) present	246 (54.5)	410 (58.2)	505 (61.9)
Edentulous	26 (14.2)	67 (21.4)	78 (24.7)
Denture			
Satisfactory	157 (85.8)	246 (78.6)	238 (75.3)
Unsatisfactory	29 (9.1)	56 (10.6)	115 (17.5)
No denture present	291 (90.9)	470 (89.4)	541 (82.5)
Oral cleanliness			
Satisfactory	247 (51.8)	348 (45.1)	257 (28.7)
Unsatisfactory	230 (48.2)	424 (54.9)	637 (71.3)
Pain			
Absent	448 (93.9)	718 (93.0)	783 (87.6)
Present	29 (6.1)	54 (7.0)	111 (12.4)

*Note*: Value are numbers (percentages) unless otherwise stated.

### 3.4 Type and frequency of dental treatment provided

Table 5 shows the type of treatment provided by the dentists. A combination of diagnostic services and preventive services was the most frequent type of care provided. This course of care accounted for 40.8%, 44.0%, 40.6% of care delivered in 2017, 2018, and 2019, respectively. Collectively, these comprised an average of 71.9% of total counts of treatment provided across the 3‐year period (Table 6). Diagnostic + preventive + restorative services were the second most frequently provided services and accounted for 21.4%, 18.3%, 21.3% of care provided in 2017, 2018, 2019, respectively. Periodontics, oral surgery (including dental extractions), and endodontics were the least utilized items ranging from 0.1% to 2.8% of the total prescribed items. The general (900) service category consists of travel to provide services, palliative review and other miscellaneous adjunctive services. A detailed breakdown of each treatment combination per course of care for each calendar year is included in Appendix 3.

**TABLE 5:** Most prescribed treatment combinations per course of care for each calendar year.

Most prescribed treatment combinations per course of care per each calendar year	2017	2018	2019
Diagnostic services only	25 (7.4)	36 (6.9)	33 (6.1)
Diagnostic + preventive services	137 (40.8)	228 (44.0)	221 (40.6)
Diagnostic + preventive + restorative services	72 (21.4)	95 (18.3)	116 (21.3)
Diagnostic + preventive + general services	19 (5.7)	28 (5.4)	27 (5.0)
Diagnostic + restorative + general services	13 (3.9)	20 (3.9)	22 (4.0)
Diagnostic + prosthetic + general services	13 (3.9)	29 (5.6)	24 (4.4)
Total	83.1	84.1	81.4

*Note*: Value are numbers (percentages) unless otherwise stated.

**TABLE 6:** Summary of total count per service category per calendar year.

Service category	2017	2018	2019	Total
Diagnostic (000)	333 (40.1)	507 (38.5)	536 (38.2)	1376
Preventive (100)	277 (33.3)	434 (33.0)	457 (32.5)	1168
Periodontic (200)	9 (1.1)	10 (0.8)	40 (2.8)	59
Oral surgery (300)	14 (1.7)	23 (1.7)	20 (1.4)	57
Endodontic (400)	1 (0.1)	1 (0.1)	1 (0.1)	3
Restorative (500)	108 (13.0)	119 (9.0)	186 (13.2)	413
Prosthetic (700)	32 (3.9)	111 (8.4)	47 (3.3)	190
General (900)	57 (6.9)	111 (8.4)	117 (8.3)	285
Total	831	1316	1404	3551

*Note*: Value are numbers (percentages).

## 4 DISCUSSION

Frequency of dental service usage declines as people age, even more for those with physical and cognitive disabilities living in residential care.^5^, ^15^, ^16^, ^17^ While limited literature is available reporting the dental service usage for the older people in residential care; the usage amongst the community dwelling old people were also low, especially for those with low‐income or without dental insurance.^18^ This study demonstrated that a large number of older people in residential care facilities could be assessed and treated through a domiciliary service pathway where they would not be seen otherwise. They received core services including diagnostic and preventive services as a means to maintain their oral health and their natural dentition. Few required more complex service such as oral surgery and emergency endodontic services.

Based on this cross‐sectional analysis of the *Reach‐OHT* database from 2017 to 2019, we found more than half of the participants had decay present (54.5%–61.9%). Close to half of the participants had a changes/unhealthy gum & soft tissue, indicative of the presence of gum diseases (40.9%–53.8%). Overall, over half of the residents scored an unsatisfactory oral cleanliness score (48.2%–71.3%). This could be due to factors such as the time of assessment; degree of oral hygiene care provided by staff, residents refusal of oral hygiene care and residents who were unable to conduct their own personal care but were still identified as independent. A large proportion of participants seen each year by *Reach‐OHT* were referred to a dentist based on their OHAT outcomes (63.4%–71.0%). Of those seen by a dentist, many required diagnostic services and preventive services. About two‐third of those seen by a dentist were able to complete the proposed treatment plan. The *Reach‐OHT* model of care appeared to fulfill the service gap when addressing access barriers to older people in residential care.

The prevalence of untreated decay and/or gum diseases, associated with an unsatisfactory oral cleanliness of older people living in residential aged care facilities, is frequently reported in the literature.^6^, ^12^, ^19^, ^20^, ^21^, ^22^, ^23^, ^24^ There is, however, little reported on what type of dental treatments people in residential care require.^10^, ^11^, ^22^, ^25^ The *Reach‐OHT* service found that diagnosis of dental diseases and treatment planning with adjunct preventive therapy were the predominant combination of treatments required. This is consistent with other studies where domiciliary dental services were provided in services not limited to residential care.^8^, ^18^, ^25^, ^26^, ^27^ It is worth noting that while 9%−13% of participants required restorative therapy, few required oral surgery, endodontic or prosthetic treatments. This raises an interesting point worthy of discussion—how do we best utilize the scope of practice of our oral health practitioner workforce in a team approach to care?

*Reach‐OHT* showed that it was possible to provide a domiciliary service to support a large number of people who are living in residential care. Oral health therapists were able to identify those people who may benefit from being seen by a dentist. Given that most people had poor oral health, it was not a surprise that the dentists provided a range of diagnostic and preventive services as a first approach to treatment. *Reach‐OHT* also demonstrated that with adequate resources, in particular the organization of dental visits and supply of portable equipment, many older people were able to have a complete course of care provided by dentists. In the Australian context, it is an important observation that the dentists working predominately in private practice were willing to be involved in the *Reach‐OHT* model of care. We believe that the key to this was to have a system that triaged those residents requiring care, organizing the dental visits, and supplying the necessary equipment.

The *Reach‐OHT* model of care and its governance structure were established to remove the commonly described barriers as identified in the literature.^10^, ^11^, ^16^, ^22^, ^25^, ^26^, ^28^, ^29^, ^30^, ^31^ As a public‐funded service, eligible participants were exempt from out‐of‐pocket payment and could therefore elect to receive or not receive the treatments recommended, free from potential financial burden. Dentists were remunerated in accordance with local policy. Other benefits of this domiciliary program were that patients did not have to travel; their dental assessment and treatments were provided in a familiar environment; and their care management records were updated at the facility. For the dentists, there was less down time between each patient; no start‐up costs for equipment procurement; readily available infrastructure within residential care facilities; and lastly minimization of anxiety and uncertainties for dentists working in a foreign environment by having a dedicated program coordinator.

The main strength of this study was that the majority of older people who were approached agreed to be seen by *Reach‐OHT* and the database captured everyone who was seen. Oral health status was assessed using a validated tool (OHAT) and allowed recording of data on the type of dental services provided by dentists. There were, however, some limitations. It is possible that the dental treatment plan proposed by dentists differed from what may have been provided in a conventional dental setting; technology and materials used may have influenced the type of treatment that dentists were willing to provide safely and confidently. Although we did not formally capture the view of our dentists; they did seem to became more comfortable providing treatment as they became familiar with both the culture and environment of the facility they were working in, which is consistently reported in other studies.^9^, ^30^ Our analysis of the type of treatments did not take into consideration the diversity of ethnicities, cultures, values, and beliefs of the participants or their surrogate decision makers but these factors may have had an impact on their decision to consent and proceed with treatment.^8^, ^32^ In this descriptive study design, it is also not possible to determine if the *Reach‐OHT* model of care resulted in better oral health, or oral health‐related function and quality of life compared to usual care.

In conclusion, this study contributes to the understanding and knowledge around the provision of domiciliary dental services in residential care. More dental practitioners should be encouraged to take action to improve awareness and satisfy the demand for outreach/domiciliary dental services as there are only a small percentage of dental practitioners or institutions who have implemented such services. As the vast majority of services provided were diagnostic, preventive and restorative care, the feasibility of utilizing the skill set of the entire dental team should also be explored.

## CONFLICT OF INTEREST STATEMENT

The authors whose names are listed under ‘Authors’ certify that they have no affiliation with or involvement in any organization or entity with any financial interest or non‐financial interest in the subject matter or material discussed in this manuscript.

## APPENDIX 1 ORAL HEALTH ASSESSMENT TOOL (OHAT)

### 1.1

Each participant will receive an oral health assessment using the oral health assessment tool form in their initial face‐to‐face assessment. The oral health assessment tool (OHAT) was a component of the Best Practice Oral Health Model for Australian Residential Care study. The OHAT provides eight categorical outcomes to assess participants’ oral health, including those with dementia. The OHAT has been utilized extensively by many and has its reliability and validity rigorously analyzed in published literature. The OHAT form used in this study is provided below where TACP‐OH clinicians will complete for each TACP participants; followed by a detailed description of how each categorical outcome is determined.

**Table:**

Parameters	Description of an ideal oral health behavior
1. Ability to practice daily oral hygiene	1.1 Independent: The resident is independent without any assistance on holding a toothbrush, dispense toothpaste, in front of a mirror, to clean his/her teeth and mouth.
	1.2 Needs reminding: The resident is independent without any assistance on holding a toothbrush, dispense toothpaste, in front of a mirror, to clean his/her teeth and mouth. However, he/she is forgetful or fail to brush daily.
	1.3 Needs supervision: The resident is independent without any assistance on holding a toothbrush, dispense toothpaste, in front of a mirror, to clean his/her teeth and mouth. However, his/her teeth and mouth are cover in plaque and appeared unable to maintain his/her oral hygiene to a satisfactory level.
	1.4 Needs full assistance: The resident is dependent on others to provide full assistance to brush his/her teeth and mouth due to disability.
2. Physical dependence present affecting daily living activities	2.1 Is independent: The resident is independent without any assistance to get out of bed, to walk, to sit at a chair and carrying on daily activities.
	2.2 Is partially dependent: The resident is partially dependent with assistance including a walking stick, walking‐aid, walking frame, and wheel‐chair to get out of bed, to sit at a chair and carrying on daily activities.
	2.3 Is highly dependent: The resident is highly dependent with assistance including a trained care staff to get out of bed, to sit on a chair and carrying on daily activities.
3. Difficult and/or challenging behavioral complication(s)	3.1 None: No behavioral complication was observed during assessment.
	3.2 Will not open mouth: The resident was not able to open his/her mouth voluntarily or involuntarily.
	3.3 Grinding or chewing: The resident was grinding or chewing his/her teeth or exhibiting chewing motion involuntarily.
	3.4 Head faces down: The resident has his/her head faces down due to medical conditions including previous injury or osteoporosis and was not able to lift his/her head for assessment.
	3.5 Refuses treatment: The resident refuses assessment.
	3.6 Is aggressive: The resident was aggressive during assessment.
	3.7 Bites: The residents attempt to bite during assessment.
	3.8 Excessive head movement: The resident was not able to keep his/her head steady during assessment.
	3.9 Cannot swallow well: The resident was observed during assessment and cannot swallow fluid/food well.
	3.10 Cannot rinse and spit: The resident was observed during assessment and cannot rinse and spit.
	3.11 Will not take out denture at night: The resident will not take out denture at night; or was advised by care staff that resident does not take out denture at night.
4. Level of communications	4.1 Is able to communicate: The resident was able to communicate freely during the assessment with the assessor
	4.2 Is confused: The resident appeared to have disrupted or repetitive speech; and was unable to communicate freely with the assessor
	4.3 Is non‐verbal: The resident was not able to communicate
5. Lips	5.1 Healthy: Smooth, pink, moist
	5.2 Changed: Dry, chapped or red at corners
	5.3 Unhealthy: Swelling or lump, red/white/ulcerated, bleeding/ulcerated at corners
6. Tongue	6.1 Healthy: Normal, moist, consistent roughness, pink
	6.2 Changed: Patchy, fissured, red, coated
	6.3 Unhealthy: Patch that is red and/or white/ulcerated, swollen
7. Gums and soft tissues	7.1 Healthy: Moist, pink, smooth, no bleeding
	7.2 Changed: Dry, shiny, rough, red, swollen, sore, one ulcer/sore spot, sore under denture
	7.3 Unhealthy: Swollen, bleeding, ulcers, white/red patches, generalized redness under dentures
8. Saliva	8.1 Healthy: Moist, tissues watery and free flowing
	8.2 Changed: Dry, sticky tissues, little saliva present
	8.3 Unhealthy: Tissues parched and red, very little/no saliva present, saliva is thick
9. Natural teeth^a^	9.1 Healthy: No decayed or broken teeth or roots
	9.2 Changed: 1–3 decayed or broken teeth/roots
	9.3 Unhealthy: Four or more decayed or broken teeth/roots
10. Denture^b^	10.1 Healthy: No broken areas or teeth, worn regularly
	10.2 Changed: One broken area or tooth
	10.3 Unhealthy: Two or more broken areas or teeth
11. Oral cleanliness	11.1 Healthy: Clean and no food part icicles or tartar in mouth or on denture(s)
	11.2 Changed: Food debris, tartar, plaque 1–2 areas of mouth, or on small area of denture(s)
	11.3 Unhealthy: Unchewed food particles, tartar, plaque most areas of mouth, or on most of denture(s)
12. Pain	12.1 Healthy: No behavioral, verbal or physical signs of dental pain
	12.2 Changed: Verbal and/or behavioral signs of pain such as pulling at face, chewing lips, not eating, changed behavior
	12.3 Unhealthy: Physical pain signs including swelling of cheek or gum, broken teeth, ulcers as well as verbal and/or behavioral signs such as pulling at face, not eating, changed behavior.
13. Referral	13.1 Required
	13.2 Not required

^a^ For a person with no natural dentition; the score in ‘Natural teeth’ will be 0.

^b^ For a person without denture; the score in ‘Denture’ will be 0.

## APPENDIX 2 Commonly prescribed Department of Veterans’ Affairs schedule of fee for dental services item codes used in the outreach dental services by dentists.

### 2.1

**Table:**

Service category	Item code	Description
Diagnostic (000)	011	Comprehensive oral examination
	013	Limited examination
	014	Consultation (dental prosthetist only)
	022	Intra‐oral radiograph
	037	Panoramic radiograph
Preventive (100)	111	Removal of plaque and/or stain
	114	Removal of calculus—first visit
	115	Removal of calculus—second visit
	121	Topical application of remineralizing agent
	141	Oral hygiene instruction
	161	Fissure sealing—per tooth
	165	Desensitizing procedure—per visit
Restorative (500)	511‐513	Metallic restoration – 1 to 3 surfaces
	521–523	Adhesive restoration – 1 to 3 surfaces—anterior
	531–533	Adhesive restoration – 1 to 3 surfaces—posterior
	572	Provisional restoration

## APPENDIX 3 Most prescribed item number per course of care by patient per calendar year.

### 3.1

**Table:**

2017 Most prescribed item number per course of care by patient			
	Frequency	Percent	Cumulative Percent
1. Diagnostic + preventive services	137	40.8	40.8
2. Diagnostic + preventive + restorative services	72	21.4	62.2
3. Diagnostic service only	25	7.4	69.6
4. Diagnostic + preventive + general services	19	5.7	75.3
5. Diagnostic + restorative + general services	13	3.9	79.2
6. Diagnostic + prosthetic + general services	13	3.9	83.0
7. Other 18 combination of services (<5 total counts per each combination)	57	16.9	100.0
Total	336	100.0	

2018 Most prescribed item number per course of care by patient			
	Frequency	Percent	Cumulative Percent
1. Diagnostic + Preventive Services	228	44.0	44.0
2. Diagnostic + preventive + restorative services	95	18.3	62.4
3. Diagnostic service only	36	6.9	69.3
4. Diagnostic + prosthetic + general services	29	5.6	74.9
5. Diagnostic + preventive + general services	28	5.4	80.3
6. Diagnostic + restorative + general services	20	3.9	84.2
7. Other 23 combination of services (<5 total counts per each combination)	82	15.9	100.0
Total	518	100.0	

2019 Most prescribed item number per course of care by patient			
	Frequency	Percent	Cumulative Percent
1. Diagnostic + Preventive Services	221	40.6	40.6
2. Diagnostic + preventive + restorative services	116	21.3	61.9
3. Diagnostic service only	33	6.1	68.0
4. Diagnostic + preventive + general services	27	5.0	73.0
5. Diagnostic + prosthetic + general services	24	4.4	77.4
6. Diagnostic + restorative + general services	22	4.0	81.4
7. Other 23 combination of services (<15 total counts per each combination)	86	18.6	100.0
Total	544	100.0	
